# Network Integrative Genomic and Transcriptomic Analysis of Carbapenem-Resistant Klebsiella pneumoniae Strains Identifies Genes for Antibiotic Resistance and Virulence

**DOI:** 10.1128/mSystems.00202-19

**Published:** 2019-05-21

**Authors:** Muyoung Lee, Naina Adren Pinto, Chan Yeong Kim, Sunmo Yang, Roshan D’Souza, Dongeun Yong, Insuk Lee

**Affiliations:** a Department of Biotechnology, College of Life Science and Biotechnology, Yonsei University, Seoul, South Korea; b Department of Laboratory Medicine and Research Institute of Bacterial Resistance, Yonsei University College of Medicine, Seoul, South Korea; c Brain Korea 21PLUS Project for Medical Science, Yonsei University College of Medicine, Seoul, South Korea; d J. Craig Venter Institute, Rockville, Maryland, USA; e Department of Biomedical Systems Informatics, Yonsei University College of Medicine, Seoul, South Korea; Institute for Genomics & Systems Biology

**Keywords:** *Klebsiella pneumoniae*, antimicrobial resistance, carbapenem, gene network, virulence

## Abstract

Global increases in the use of carbapenems have resulted in several strains of Gram-negative bacteria acquiring carbapenem resistance, thereby limiting treatment options. Klebsiella pneumoniae is a common carbapenem-resistant pathogenic bacterium that is widely studied to identify novel antibiotic resistance mechanisms and drug targets. Antibiotic-resistant clinical isolates generally harbor many genetic alterations, and the identification of responsible mutations would provide insights into the molecular mechanisms of antibiotic resistance. We propose a method to prioritize mutated genes responsible for antibiotic resistance on the basis of expression changes in their local subnetworks, hypothesizing that mutated genes that show significant expression changes among the corresponding functionally associated genes are more likely to be involved in the carbapenem resistance. For network-based gene prioritization, we developed KlebNet (www.inetbio.org/klebnet), a genome-scale cofunctional network of K. pneumoniae genes. Using KlebNet, we reconstructed the functional modules for carbapenem resistance and virulence and identified the functional association between antibiotic resistance and virulence. Using complementation assays with the top candidate genes, we were able to validate a novel gene that negatively regulated carbapenem resistance and four novel genes that positively regulated virulence in Galleria mellonella larvae. Therefore, our study demonstrated the feasibility of network-based identification of genes required for antibiotic resistance and virulence of human-pathogenic bacteria.

**IMPORTANCE** Klebsiella pneumoniae is a major bacterial pathogen that causes pneumonia and urinary tract infections in human. K. pneumoniae infections are treated with carbapenem, but carbapenem-resistant K. pneumoniae has been spreading worldwide. We are able to identify antimicrobial-resistant genes among mutated genes of the antibiotic-resistant clinical isolates. However, they usually harbor many mutated genes, including those that cause weak or neutral functional effects. Therefore, we need to prioritize the mutated genes to identify the more likely candidates for the follow-up functional analysis. For this study, we present a functional network of K. pneumoniae genes and propose a network-based method of prioritizing the mutated genes of the resistant clinical isolates. We also reconstructed the network-based functional modules for carbapenem resistance and virulence and retrieved the functional association between antibiotic resistance and virulence. This study demonstrated the feasibility of network-based analysis of clinical genomics data for the study of K. pneumoniae infection.

## Abstract (precis)

Klebsiella pneumoniae is a major bacterial pathogen that causes pneumonia and urinary tract infections in human. K. pneumoniae infections are treated with carbapenem, but carbapenem-resistant K. pneumoniae has been spreading worldwide. We are able to identify antimicrobial-resistant genes among mutated genes of the antibiotic-resistant clinical isolates. However, they usually harbor many mutated genes, including those that cause weak or neutral functional effects. Therefore, we need to prioritize the mutated genes to identify the more likely candidates for the follow-up functional analysis. For this study, we present a functional network of K. pneumoniae genes and propose a network-based method of prioritizing the mutated genes of the resistant clinical isolates. We also reconstructed the network-based functional modules for carbapenem resistance and virulence and retrieved the functional association between antibiotic resistance and virulence. This study demonstrated the feasibility of network-based analysis of clinical genomics data for the study of K. pneumoniae infection.

## INTRODUCTION

Carbapenem-resistant Klebsiella pneumoniae is an important opportunistic bacterial pathogen that causes pneumonia and urinary tract infections with a mortality rate of around 40% (1). Carbapenem is known as an antibiotic of last resort because of its broad spectrum of activity among β-lactam antibiotics and resistance to most β-lactamases produced by bacteria (2). However, carbapenem-resistant K. pneumoniae strains have been spreading worldwide. The U.S. CDC has reported that more than 9,000 health care-associated infections are caused by carbapenem-resistant Enterobacteriaceae each year (Antibiotic resistance threats in the United States, 2013), and K. pneumoniae is isolated most commonly in strains of carbapenem-resistant Enterobacteriaceae (3). The percentages of invasive infections by carbapenem-resistant K. pneumoniae have been reported to be ≥50% in Greece, ≥25% in Italy, and <1% in Scandinavian countries (Surveillance of antimicrobial resistance in Europe, 2013). In Sri Lanka and Western Australia, there have been waves of infections by strains of carbapenem-resistant K. pneumoniae (4, 5). The World Health Organization identified carbapenem-resistant *Klebsiella* species as being among the most urgent problems in their first global report on antimicrobial resistance in 2014 (6). We focus on meropenem, a member of the carbapenem class of antibiotics, in this study.

Resistance mechanisms for carbapenem-nonsusceptible K. pneumoniae isolates, including carbapenemase (such as KPC, NDM, etc.), upregulation of efflux pumps, extended-spectrum β-lactamases accompanied by porin loss, and hyperproduction of AmpC β-lactamase, have been reported previously (7). However, some cases of carbapenem resistance in K. pneumoniae cannot be explained using the mechanisms mentioned above. In this study, we collected two consecutive K. pneumoniae isolates retrospectively from a single patient. The strain was initially meropenem susceptible but became resistant after the meropenem treatment. We could not explain the mechanisms conferring carbapenem resistance to the strain. Hence, further identification of K. pneumoniae genes responsible for carbapenem resistance is required to provide important clues for solving the problems of antibiotic resistance.

Mutated genes of the antibiotic-resistant clinical isolates are putative genes hypothesized to be responsible for the antimicrobial resistance phenotype. However, antibiotic-resistant strains isolated from patients usually harbor many mutated genes, including those that cause weak or neutral functional effects. Therefore, we need to prioritize the mutated genes to identify the more likely candidates, which can facilitate follow-up functional analysis. In this report, we propose a method of network integrative genomic and transcriptomic analysis for prioritizing the mutated genes of carbapenem-resistant clinical isolates.

## RESULTS

### K. pneumoniae strains with acquired carbapenem resistance show genomic and transcriptomic alterations for many genes.

Meropenem is a member of the carbapenem class of β-lactam antibiotics used to treat K. pneumoniae infections. We used two strains of K. pneumoniae clinical isolates from a single patient who had undergone meropenem treatment, namely, YMC2014/2/R777 and YMC2014/3/P345, which we refer to as K26 and K56, respectively, in this paper, that were isolated before and after the meropenem administration, respectively. In K. pneumoniae, two major outer membrane porins, OmpK35 and OmpK36, play roles in both antimicrobial resistance and virulence (8), and both K26 and K56 have a mutation in *ompK35* (Table 1). Porin loss is a well-known mechanism for the acquisition of carbapenem resistance in K. pneumoniae (9). The presence of a defective *ompK35* gene in susceptible strain K26 can be explained by a previous report where a single deletion of *ompK35* (Δ*ompK35*) had no significant effect on antibiotic resistance (8). Although both strains have genes for β-lactamase, they differ in their susceptibility to carbapenem. There was no known carbapenemase gene present in these clinical isolates. An increase in the meropenem MIC, from 0.5 μg/ml to ≥32 μg/ml, was observed in K56 compared with K26.

**TABLE 1:** β-Lactamase and porin gene profiles of K. pneumoniae strains used in this study

Strain	β-Lactamase genes	Mutation in porin genes	
		OmpK35 (KPHS_18380)	OmpK36 (KPHS_37010)
K26	*bla*_SHV-12_, *bla*_DHA-1_, *bla*_LEN-11_	1-bp deletion (54/435 nt),^*a*^ C insertion (398/435 nt)	No mutation
K56	*bla*_SHV-12_, *bla*_DHA-1_, *bla*_LEN-11_	1-bp deletion (54/435 nt), C insertion (398/435 nt)	No mutation

a nt, nucleotide.

The acquisition of antimicrobial resistance is attributable to some genetic alterations that occurred in the resistant strains. To identify genetic variations of the antibiotic-resistant strains, we conducted whole-genome sequencing for the susceptible strain (K26) and the resistant strain (K56) and performed a mutation calling analysis using *breseq* software (v. 0.33.1) (10), which identifies mutated nucleotide sequences based on sequence read alignment on a reference genome. We used a two-step procedure of mutation calling using the K56 genome rather than the K26 genome, because a completely assembled genome for the K26 is not yet available. First, we aligned sequence reads from K26 to a completely assembled genome of K. pneumoniae HS11286 to identify mutations on the K26 genome compared with HS11286 genome. Then, using the APPLY function of gdtools in *breseq* software, we applied identified mutations (described in GenomeDiff format) of K26 to the HS11286 genome to generate a “pseudo-K26” genome. Alignment of sequence reads from K56 on the pseudo-K26 genome allowed us to identify 16 deleted genes (representing one large deletion) and 14 mutated genes in K56 compared with susceptible parent strain K26 (see Table S1 in the supplemental material). Both the deleted genes and the mutated genes are referred to simply as “mutated” genes in this article.

10.1128/mSystems.00202-19.1 TABLE S1 Mutations occurred in the resistant strain (K56; *in vivo* isolate). *P* value data represent significance of the expression changes for the local subnetwork of each mutated or deleted gene using two-sample K-S testing. Download Table S1, XLSX file, 0.01 MB. Copyright © 2019 Lee et al. 2019 Lee et al. This content is distributed under the terms of the Creative Commons Attribution 4.0 International license.

Genomic alterations cause changes in transcriptome profiles in antibiotic-resistant strains. We measured gene expression levels via RNA sequencing for the same strains. The raw sequence reads were aligned to the reference genome using STAR aligner (11), and relative changes in gene expression between the susceptible parent strain (K26) and the resistant strain (K56) were quantified using DESeq2 software (12). We found 34 and 7 genes that were upregulated and downregulated significantly (fold change, >4; *P < *0.01), respectively, in the resistant strain (K56) compared with the parent susceptible strain (K26) (Table S2).

10.1128/mSystems.00202-19.2 TABLE S2 (A) Expression fold changes (FC) in the resistant strain K56 *in vivo* isolate. (B) Upregulated genes in the resistant strain K56 *in vivo* isolate (FC value, >4; *P* value, <0.01). Download Table S2, XLSX file, 0.3 MB. Copyright © 2019 Lee et al. 2019 Lee et al. This content is distributed under the terms of the Creative Commons Attribution 4.0 International license.

Gene set enrichment analysis revealed that the set of 34 upregulated genes in strain K56 were significantly associated with the GO biological process terms “response to antibiotics,” “lipid A biosynthetic process,” “lipopolysaccharide (LPS) biosynthetic process,” “lipid A metabolic process,” “4-amino-4-deoxy-alpha-l-arabinopyranosyl undecaprenyl phosphate biosynthetic process,” and “lipid metabolic process” (*P < *0.01 by Fisher’s exact test; overlap of at least two genes) and were involved in the formation of the outer membrane and its function. These results may suggest that trajectories of molecular evolution for the acquisition of a meropenem-resistant strain (K56) in the patient changed the activity of effector genes, resulting in increased bacterial outer membrane biosynthesis. Since most antibiotics target intracellular processes, they must be able to penetrate the bacterial cell envelope (13). In Gram-negative bacteria such as K. pneumoniae, the outer membrane is mainly composed of LPS, which provides a formidable barrier against xenobiotics. Therefore, bacterial cells may often enhance outer membrane formation to counter antibiotics that decrease membrane permeability.

### Network-based strategy to prioritize mutated genes for acquired carbapenem resistance.

**FIG 1:**
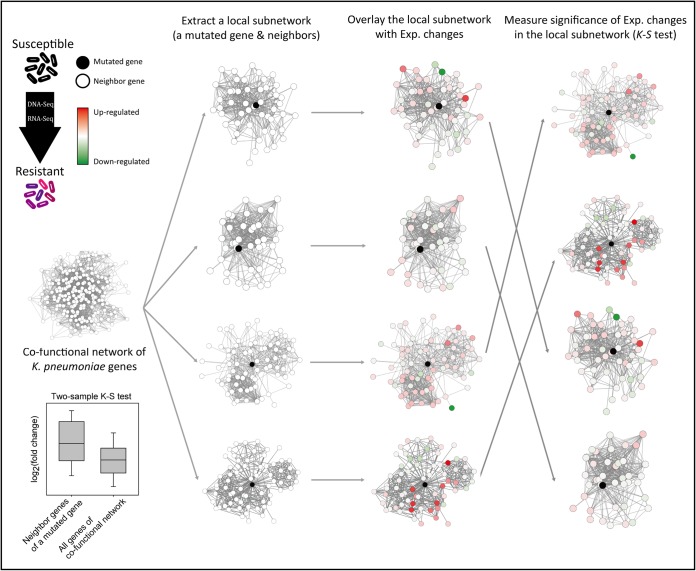
Schematic overview of network-based prioritization of mutated genes of clinical isolates for antibiotic resistance. Based on the DNA and RNA sequencing profiles of the isolated resistant strains and a susceptible parent strain, we identified genetic alterations and expression (Exp.) changes for all K. pneumoniae genes. Using a cofunctional network of K. pneumoniae genes, we extracted local subnetworks (a mutated gene and its neighbor genes) and overlaid them with expression fold changes. We then measured the significance of the expression changes [log_2_(fold change)] for network neighbors of each mutated gene by the use of the two-sample Kolmogorov-Smirnov (K-S) test. The four examples of local subnetworks given are ordered from the top row by the *P* values determined by the K-S test.

As mentioned above, antibiotic-resistant clinical isolates such as K56 usually contain many genomic alterations, and it is unlikely that all the mutated genes are involved in the antibiotic resistance. Thus, we need to prioritize the mutated genes to facilitate the discovery of novel genes for antibiotic resistance. We hypothesized that if genetic alterations in a gene resulted in antibiotic resistance, the mutations might cause significant expression changes among the functionally associated genes that might comprise a subsystem responsible for the acquired antibiotic resistance phenotype. Because cofunctional networks connect functionally associated genes (14), we can represent subsystems as a local subnetwork that is composed of a mutated gene and its neighbors in the cofunctional network. We may then prioritize the mutated genes in accordance with the significance of expression changes in the local subnetworks. Here, to avoid bias from the different functional effects of the mutations, we did not use expression changes of the mutated genes for the statistical analysis. We measured the significance of the expression changes based on log_2_(fold change) for the local subnetwork of each mutated gene using the two-sample Kolmogorov-Smirnov (K-S) test (Fig. 1).

### Construction of KlebNet for network-based study of K. pneumoniae genes.

**FIG 2:**
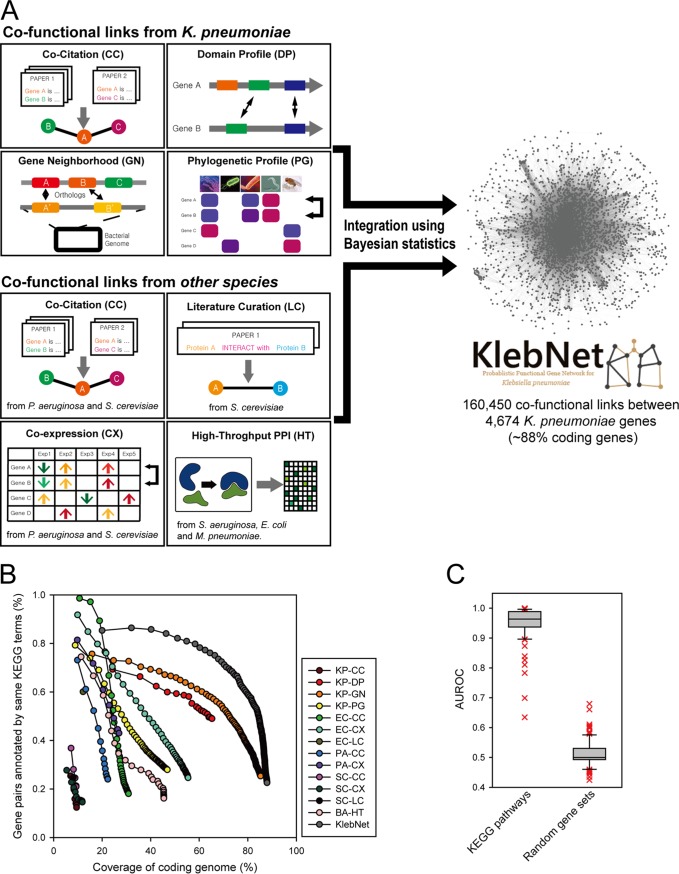
Overview of KlebNet. (A) Network construction by cofunctional link inferences from diverse types of omics data and species and their integration. (B) Assessment of cofunctional links of KlebNet and individual component networks based on proportions of gene pairs annotated by same KEGG pathway terms, measured for each bin of 1,000 links from the most confident one. (C) Assessment of prediction efficacy of KlebNet based on retrieval rate of known pathway genes for individual KEGG pathways and random gene sets with the same size, measured by area under the receiver operating characteristic curve (AUROC) scores.

To conduct a network-based study of K. pneumoniae genes such as gene prioritization for the acquired antibiotic resistance, we constructed KlebNet, a genome-scale cofunctional network (14) of K. pneumoniae genes, by machine learning and integration of diverse types of omics data using Bayesian statistics (Fig. 2). Peer-to-peer relations between K. pneumoniae genes that were likely to participate in the same biological process were inferred by the following approaches: cocitation (15, 16) of K. pneumoniae genes across PubMed abstracts, mutual association between protein domain profiles (DP) (17) between K. pneumoniae genes, association of genomic context information between K. pneumoniae genes by gene neighborhood (GN) (18), and phylogenetic profiles (PG) (19, 20). In addition to the information derived from K. pneumoniae genes, we also inferred functional links by orthology-based transfer of cofunctional or protein-protein interactions from other microbial species (21), including Escherichia coli, Pseudomonas aeruginosa, Saccharomyces cerevisiae, Mycoplasma pneumoniae, and Staphylococcus aureus. We were able to construct 13 networks from various data sources (Table 2). Those 13 component networks were then integrated into a single network by the use of a Bayes statistical framework (22). Methods of inferring cofunctional links from each data source are described in Materials and Methods. The KlebNet integrated network covers approximately 88% of the 5,316 coding genes of K. pneumoniae strain HS11286 with 160,450 cofunctional links. Information regarding the 13 component networks and KlebNet is available from a public Web server (www.inetbio.org/klebnet).

**TABLE 2:** Thirteen component networks of KlebNet^*a*^

Code	Description	No. of genes	No. of links
KP-CC	Inferred links by cocitation across PubMed Central articles for K. pneumoniae biology	504	5,505
KP-DP	Inferred links by correlation of protein domain profiles of K. pneumoniae coding genes	3,470	17,402
KP-GN	Inferred links by neighborhood of K. pneumoniae orthologs in bacterial genomes	4,533	94,499
KP-PG	Phylogenetic profiling and coinheritance patterns of K. pneumoniae ortholog genes across organisms	2,492	44,496
EC-CC	Associalogs of inferred links by cocitation across PubMed Central articles for E. coli biology	1,641	30,464
EC-CX	Associalogs of inferred links by coexpression across gene expression profiles in the GEO database for E. coli	2,946	42,489
EC-LC	Associalogs of bacterial protein-protein interactions derived from literature curation of small-scale analysis for E. coli	646	840
PA-CC	Associalogs of inferred links by cocitation across PubMed Central articles for P. aeruginosa biology	1,187	12,181
PA-CX	Associalogs of inferred links by coexpression across gene expression profiles in the GEO database for P. aeruginosa	1,433	6,356
SC-CC	Associalogs of inferred links by cocitation across PubMed Central articles for S. cerevisiae biology	495	4,294
SC-CX	Associalogs of inferred links by coexpression across gene expression profiles in the GEO database for S. cerevisiae	630	9,513
SC-LC	Associalogs of bacterial PPI derived from literature curation of small-scale analysis for S. cerevisiae	435	896
BA-HT	Associalogs of bacterial PPI derived from high-throughput data of E. coli, S. aureus, and M. pneumoniae	2,415	23,493
KlebNet	Integrated network	4,674	160,450

a KP, K. pneumoniae; EC, E. coli; PA, P. aeruginosa; SC, S. cerevisiae; BA, multiple bacterial species; CC, cocitation; DP, domain profile; GN, gene neighborhood; PG, phylogenetic profiles; CX, coexpression; LC, literature-curated protein-protein interactions; HT, high-throughput protein-protein interactions.

Although KlebNet performs network searches on the basis of a K. pneumoniae subsp. *pneumoniae* HS11286 gene network, the network search is also compatible with four other K. pneumoniae strains of major importance: NTUH-K2044, MGH 78578, KCTC 2242, and Ecl8. For example, if users submit gene names for strain Ecl8, the webserver uses orthologous HS11286 strain genes for the network search. All candidate genes are reported by both HS11286 gene names and Ecl8 gene names. For orthologous gene mapping of major K. pneumoniae strains, we used bidirectional best hits according to BLASTP (Basic Local Alignment Search Tool for Proteins).

### KlebNet is highly predictive with respect to diverse pathways in K. pneumoniae.

To ensure the efficacy of network-based prioritization of mutated genes, we assessed the quality of KlebNet. To assess the precision of prediction of cofunctional gene pairs by KlebNet, we used test data that were independent of those used for training the network to avoid circularity. We compiled gene pairs from KEGG pathways (23), independent annotations from UniProt Gene Ontology biological processes (UniProt-GOBP) (24), and the MetaCyc metabolic pathways (25) that were used for training KlebNet (see Materials and Methods). Consistent with their independent origin, the test data derived from KEGG pathway annotations overlapped only 19% of the gene pairs used for network training. In the network assessment performed on the basis of the test data, we observed higher precision of cofunctional gene pairs from the integrated KlebNet than were seen with the component networks derived from individual data sources, which indicates that integration of diverse omics data effectively improved the quality of KlebNet (Fig. 2B).

Next, we evaluated the capability of KlebNet to predict pathways in K. pneumoniae by measuring the efficacy of retrieval of known genes for each KEGG pathway. If genes known for a pathway are effectively retrieved by network connections to other member genes of the same pathway, novel genes for the pathway are also likely to be retrieved by the network. We therefore measured the rate of retrieval of known genes for each of 93 KEGG pathway terms that have no fewer than five member genes, using receiver operating characteristic (ROC) analysis. The ROC curve behavior can be summarized as a score, the area under the ROC curve (AUC), which would be close to 0.5 for a network of randomly selected genes and would approach 1 for a perfect network. We found that efficacy of KlebNet retrieval for the KEGG pathway terms was significantly higher than that for random gene sets (*P < *2.2e−16 [Wilcoxon rank sum test]) (Fig. 2C). These results indicate that KlebNet accurately mapped cofunctional links between K. pneumoniae genes and would also be able to predict novel genes for a pathway based on network links to the genes already known to the same pathway.

### KlebNet-based prioritization of mutated genes for the acquired carbapenem resistance.

Using the computationally validated cofunctional network of K. pneumoniae genes, KlebNet, and the network-based strategy summarized in Fig. 1, we prioritized mutated genes of the strain with acquired carbapenem resistance (K56). Mutations may not influence expression levels of all local neighbor genes. Thus, we sorted the local neighbor genes for each mutated gene by descending order of edge weight (log-likelihood score [LLS]) and then repeated the K-S test with the cumulative number of closest neighbor genes from 5 to 100 in increments of 5. For each mutated gene, we chose a set of neighbor genes with the most significant *P* value by K-S test. We excluded genes that were predicted to encode putative prophage tail, sheath, and capsid proteins from the candidate list because their sequences differ among strains. For a typical significance threshold (*P < *0.05), we were able to obtain 15 candidate genes that are possibly involved in carbapenem resistance (Table S1).

### KlebNet retrieved functional associations between antibiotic resistance and virulence.

**FIG 3:**
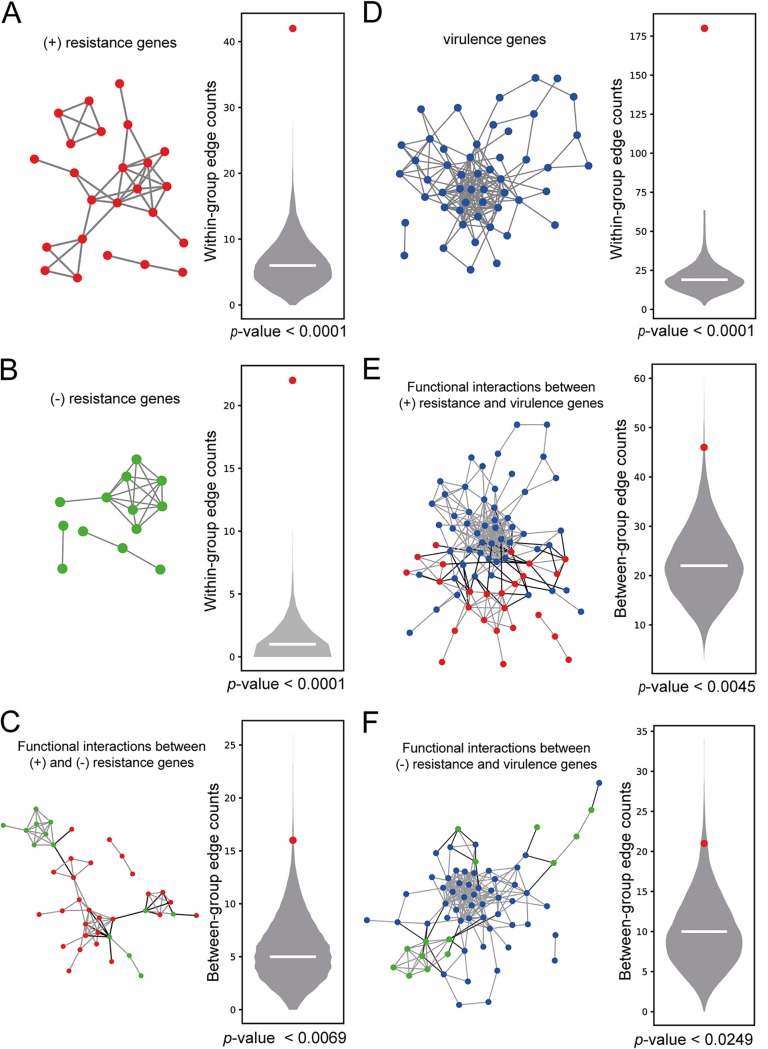
Within-group and between-group connectivity in KlebNet for genes involved in carbapenem resistance and virulence. (A) Analysis of within-group connectivity among positive (+)-resistance genes (34 genes upregulated in K56). The null model of the within-group edge count was generated by the use of 10,000 random sets of 34 genes. The horizontal line in the violin plot indicates the median, and the red dot indicates the within-group edge count for the positive-resistance genes. The *P* value was determined on the basis of a permutation test. (B) Analysis of within-group connectivity among negative (−)-resistance genes (15 genes that were mutated in meropenem-resistant strain K56). A significance test was performed as described for panel A. (C) Analysis of connectivity between 15 negative-resistance genes and 34 positive-resistance genes. KlebNet edges for within-group connections are indicated by shading in the network. The null model of the between-group edge count was generated by the use of 10,000 paired random sets, one set of 15 random genes, and one set of 34 random genes. The *P* value was determined on the basis of a permutation test. (D) Analysis of within-group connectivity among 59 virulence genes associated with mouse lung infections. A significance test was performed as described for panel A. (E) Analysis of connectivity between 34 positive-resistance genes and 59 virulence genes. Significance testing was performed as described for panel C. (F) Analysis of connectivity between 15 negative-resistance genes and 59 virulence genes. Significance testing was performed as described for panel C.

The set of 34 upregulated genes in meropenem-resistant strain K56 were likely to be responsible for the increased antibiotic resistance. Therefore, we refer to the 34 upregulated genes in the meropenem-resistant strains as “positive-resistance” genes. Because the meropenem resistance genes were significantly enriched for biological processes involving outer membrane formation, we tested if these genes were also functionally coherent using a within-group connectivity analysis in KlebNet. To measure the significance of the within-group connectivity for a given gene set, we generated a null distribution model of within-group edge counting using 10,000 random gene sets of the same size and examined the rank of the within-group edge count of the real gene set in the null distribution. As expected, the results reflecting network connectivity among the meropenem resistance genes were significant (*P < *0.0001 by permutation test) (Fig. 3A), which indicated that KlebNet was highly predictive for positive modulators of meropenem resistance.

Mutated genes in the meropenem-resistant strains were likely to be potentially associated with the antibiotic susceptibility of the parent strain (K26). We found that 15 mutated genes of K56 with significant expression changes in local subnetworks were functionally coherent as indicated by the significant within-group connectivity in KlebNet (Fig. 3B). Here, we refer to the group of 15 genes as “negative-resistance genes.”

As expected, there was no overlap of the 34 positive-resistance genes and 15 negative-resistance genes. However, we hypothesized that antibiotic resistance genes with different modes of action (positive and negative) were still functionally associated with each other and comprised a cellular subsystem for the regulation of antibiotic resistance. If so, two groups of genes, a group of 34 genes for positive resistance and another group of 15 genes for negative resistance, should be significantly connected by cofunctional links in KlebNet. To measure the significance of between-group connectivity for the given two gene sets, we generated a null distribution model of edge counts of 10,000 pairs of random gene sets with the same sizes (one set had 34 random genes and the other set had 15 random genes) and then examined the ranks of between-group edge counts of two real gene sets in the null distribution. We found that the data representing functional connections between the positive-resistance and negative-resistance genes were significant (*P < *0.0069 by permutation test) (Fig. 3C) but not as significant as the data representing within-group connectivity for each mode of action (see Fig. 3A and B).

Antibiotic resistance is known to be associated with a fitness cost, because reduced growth rates are often observed in antibiotic-resistant strains (26). The observed phenotypic association suggests a functional association between the genetic modules for antibiotic resistance and those for virulence. To model a functional module for the virulence of K. pneumoniae, we compiled genes from an earlier study that simultaneously screened thousands of transposon insertion mutants for fitness defects during a mouse lung infection by high-throughput sequencing (27). In this analysis, fitness defects were represented by a read count ratio of the inoculum to lung. We selected as “virulence genes” only 59 genes that showed at least 10-fold higher read counts in the inoculum for our analysis (Table S3). We found that the data from the 59 virulence genes showed significant connections among those genes (*P < *0.0001 by permutation test) (Fig. 3D).

10.1128/mSystems.00202-19.3 TABLE S3 Ratios of lung to inoculum read counts between insertions in the same gene. Highlighted genes are defined as “fitness genes” for mouse lung infection [ratio = (count_inoculum/count_lung) >10]. Download Table S3, XLSX file, 0.08 MB. Copyright © 2019 Lee et al. 2019 Lee et al. This content is distributed under the terms of the Creative Commons Attribution 4.0 International license.

Using these virulence genes, we measured the significance of association between virulence and antibiotic resistance with either positive or negative mode of action. First, we examined the gene-based overlap of 59 virulence genes and either 34 genes for positive resistance or 15 genes for negative resistance. We found no overlap of 15 negative-resistance genes and only a single gene overlap of 34 positive-resistance genes with the 59 virulence genes. Therefore, the data reflecting the gene-based overlap could not support the idea of a functional association between antibiotic resistance and virulence in K. pneumoniae. Alternatively, two functional modules were found to be associated with each other with respect to functional connections between genes from different modules. We measured the significance of functional connections between a module of 59 virulence genes and a module of 34 positive-resistance genes or a module of 15 negative-resistance genes by using null models of between-group edge counts generated as described above. We found that both the positive resistance and negative-resistance modules were significantly connected with the virulence module in KlebNet (*P < *0.0045 and *P < *0.0249, respectively, by permutation test) (Fig. 3E and F). Therefore, KlebNet was successfully used to retrieve the association between antibiotic resistance and virulence in K. pneumoniae.

### Experimental validation of prioritized mutated genes for carbapenem resistance and virulence of K. pneumoniae.

**FIG 4:**
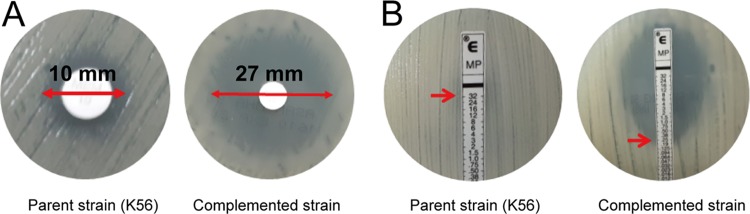
Complementation assay for resistant strains performed with *KPHS_33600*. (A) A meropenem disk diffusion assay showed an inhibitory zone diameter increase (from 10 to 27 mm) resulting from complementation of K56 with *KPHS_33600*. (B) A meropenem Etest strip assay revealed that the MICs of K56::ZpUC19 and K56::ZpUC19-*KPHS_33600* were ≥32 and 0.25 μg/ml, respectively, as indicated by the red arrows. The data were confirmed by CLSI broth dilution tests. Upon complementation, a 128-fold reduction in the MIC for the resistant strain was seen as indicated.

We chose 15 mutated genes with significant expression changes among the local subnetworks in K56 (*P < *0.05 by K-S test) for the follow-up functional analysis. We were able to complement the meropenem-resistant strains with only 11 of the 15 candidate genes (see Table S1). In the assay, mutated genes in the resistant strains were complemented with the intact gene of the susceptible parent strain (K26) to determine the changes in meropenem susceptibility. One candidate gene, K. pneumoniae *HS_33600* (*KPHS_33600*), showed increased susceptibility to meropenem as indicated by the presence of an increased inhibition zone diameter in the disk diffusion assay (Fig. 4A) and by the decreased MIC of meropenem in the Etest (Fig. 4B). The results were confirmed using two independent broth microdilution experiments.

**FIG 5:**
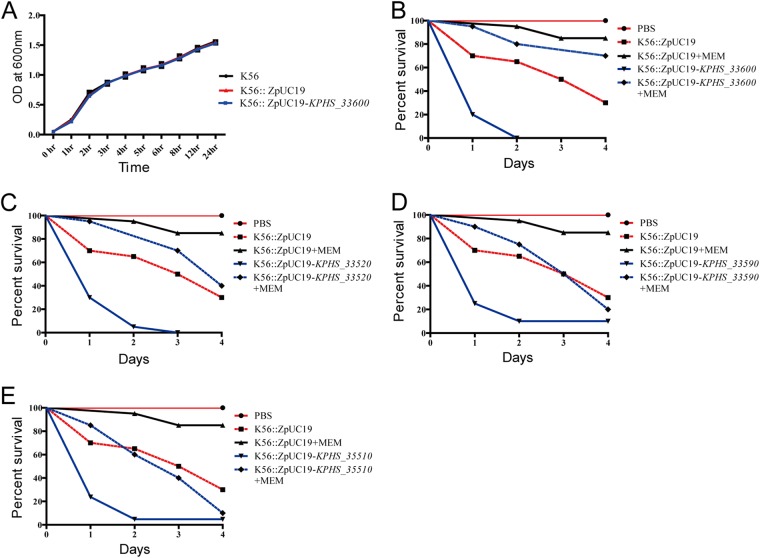
*In vitro* and *in vivo* fitness assays. For the *in vitro* fitness assay, growth assays were conducted in LB broth at 37°C with continuous shaking for the two complemented strains (K56::ZpUC19 and K56::ZpUC19-*KPHS_33600*) (A) along with the parent strain K56 with and without the ZpUC-19 empty vector, respectively. There were no differences seen between the results determined with the resistant strain and the complemented strain. For the *in vivo* fitness assay, G. mellonella larvae were infected with approximately 1 × 10^6^ CFU of bacteria. After 4 h of infection, meropenem was injected and the larvae were observed for 4 days. Data represent results of comparisons of percentages of survival between larvae infected by the parent strain (K56) and those infected by strains complemented with (B) *KPHS_33600*, (C) *KPHS_33520*, (D) *KPHS_33590*, and (E) *KPHS_35510*. MEM, meropenem.

Next, we examined whether the mutated genes could affect fitness using an *in vitro* growth assay (26). We observed no differences in growth rates in comparisons between the parent resistant strain (K56) and the complemented candidates (K56 complemented with *KPHS_33600*) in the antibiotic-free media (Fig. 5A). Although the antibiotic resistance acquired as a consequence of defects of *KPHS_33600* did not result in a fitness cost with respect to their growth *in vitro*, there could still have been a cost with respect to virulence (i.e., *in vivo* fitness). We thus examined the effects on virulence of complementation with the 11 candidate genes in Galleria mellonella larvae as described in Materials and Methods. We found that complementation with *KPHS_33600* increased the virulence of parent resistant strain K56 (Fig. 5B). Interestingly, K56 strains complemented with three additional candidate genes, *KPHS_33520* (Fig. 5C), *KPHS_33590* (Fig. 5D), and *KPHS_35510* (Fig. 5E), failed to show increased meropenem susceptibility but showed increased virulence. Therefore, we successfully validated 4 of the 11 tested candidate genes with respect to either antibiotic resistance or virulence (discovery rate, 36.4%).

## DISCUSSION

In this study, we demonstrated the feasibility of our network-based method for effective identification of genes involved in carbapenem resistance by focusing on meropenem and virulence. For network-based prioritization of K. pneumoniae genes, we constructed KlebNet, a genome-scale cofunctional network of K. pneumoniae genes. We found that KlebNet was highly predictive of genes involved in various KEGG pathways and in carbapenem resistance and virulence. KlebNet was also used to explain the fitness cost of antibiotic resistance using significant functional connections between antibiotic resistance and virulence genes. In the complementation assay of the 11 top candidate genes, we were able to identify 1 novel gene involved in carbapenem resistance and 4 novel genes involved in the virulence of K. pneumoniae.

To obtain mechanistic clues for the novel genes for the carbapenem resistance and virulence, we conducted literature surveys. *KPHS_33600*, required for both carbapenem resistance and virulence, which was annotated as a putative transmembrane transport protein by GO biological process annotation, turned out to be homologous to the genes encoding the major facilitator superfamily (MFS) transporter present in many bacteria. Although MFS transporters generally work as efflux pumps and their defects are expected to increase antibiotic susceptibility, mutated *KPHS_33600* induced antibiotic resistance, and its complementation increased antibiotic susceptibility in our study. To justify these unexpected observations, we conducted a literature survey and found multiple studies that previously reported similarly counterintuitive observations. Deletion of *efpA*, a putative efflux pump gene of Mycobacterium smegmatis, increased susceptibility to fluoroquinolones, ethidium bromide, and acriflavine but also decreased susceptibility to rifamycins, isoniazid, and chloramphenicol (28). Overexpression of a multidrug efflux pump encoded by *cmlA* in E. coli caused spectinomycin hypersensitivity (29). Overexpression of efflux pump protein genes *smeDEF* and *smeX* in Stenotrophomonas maltophilia resulted in decreased aminoglycoside resistance (30, 31). To date, there has been no clear explanation for these counterintuitive observations.

Next, we conducted a survey of the literature to look for evidence of associations with virulence for the three genes involved in fitness but not in antibiotic susceptibility. *KPHS_33520* is a putative rRNA large-subunit methyltransferase A gene. The 16S rRNA methyltransferases encoded by *rsmI* and *rsmH* have been reported to be involved in virulence in Staphylococcus aureus (32). Although we could not detect significant homologies between *KPHS_33520* genes and RsmI or RsmH proteins using BLASTP analysis, there could be another rRNA methyltransferase involved in the virulence of K. pneumoniae. *KPHS_33590* is a putative IclR family transcriptional regulator. KdgR, a member of the IclR family, has been shown to be involved in virulence in plant-pathogenic *Erwinia* spp. (33). We found significant homology between the KdgR gene and *KPHS_33590* (87% BLASTP sequence identity with an E value of 6.83e−179). *KPHS_35510* is a putative UDP galacturonate 4-epimerase gene. The K. pneumoniae MGH 78578 strain has a gene named *uge* (for “UDP galacturonate 4-epimerase”) which has been shown to be essential for virulence in septicemia and pneumonia animal models (34). Furthermore, *KPHS_33590* is an *uge* homolog (98% BLASTP sequence identity with E value = 0). In summary, we were able to identify literature evidence supporting the idea of associations of virulence with all three novel K. pneumoniae genes for *in vivo* fitness.

Given that cofunctional gene networks are available for many pathogenic microbial species, application of the proposed network-based method for prioritizing mutated genes from clinical isolates of other species should be straightforward and will facilitate our understanding of other human-pathogenic microbes. In addition, the KlebNet web server (www.inetbio.org/klebnet) would be a useful resource for the study of other biological processes involved in host-pathogen-drug interactions of K. pneumoniae.

## MATERIALS AND METHODS

### Bacterial selection, identification, and susceptibility testing.

Two K. pneumoniae clinical isolates, K26 and K56, used in this study were selected from a collection of bacteria maintained in the laboratory of Severance Hospital, Seoul, South Korea. The strains were isolated from a single patient who had undergone meropenem treatment. K26 was collected before the meropenem treatment, while K56 was collected after the treatment. The strains were identified using a matrix-assisted laser desorption ionization–time of flight mass spectrometry (MALDI-TOF MS) Biotyper CA System (Bruker Daltonik GmbH). The clonal relatedness was determined using pulse-field gel electrophoresis as previously described (35). The meropenem MIC was determined by the Etest and by CLSI disk diffusion and broth dilution protocols. P. aeruginosa ATCC 27853 and E. coli ATCC 25922 were used as control strains as recommended by the CLSI guidelines. The results were confirmed using three independent experiments.

### DNA and RNA sequencing for K. pneumoniae clinical isolates.

For genomic sequence analysis of two clinical strains, genomic DNA was extracted using a Wizard genomic DNA purification kit (Promega, Madison, WI, USA). Library preparation was carried out using a TruSeq Nano DNA sample preparation kit (Illumina, San Diego, CA, USA) per the manufacturer's protocol, and the sequencing was carried out by using an Illumina-MiSeq sequencing platform (2× 300-bp paired-end protocol).

For gene expression analysis, we isolated RNA by growing the clinical isolates to the logarithmic phase in high-osmolality LB broth at 37°C and extracting the RNA using an RNeasy minikit (Qiagen GmbH, Hilden, Germany). On-column DNA digestion was carried out using an RNase-free DNase kit (Invitrogen, Carlsbad, CA, USA). RNA concentrations were measured using a NanoDrop spectrophotometer (Thermo Fisher Scientific, Waltham, MA, USA). Sequencing library preparation was carried out using a TruSeq stranded total RNA library preparation kit (Illumina, San Diego, CA, USA). We performed RNA sequencing for two biological replicates using an Illumina NextSeq 500 sequencer (Illumina, San Diego, CA, USA) with ∼25 million paired-end reads.

### Genome data and gold standard cofunctional gene pairs used for network construction.

We developed a cofunctional network for the K. pneumoniae HS11286 strain, which has 5,867 genes. To include only protein-coding genes for the network, we excluded 88 RNA genes and 463 plasmid genes, leaving 5,316 protein-coding genes for the network construction. We constructed cofunctional networks based on machine learning approaches, which require gold standard (GS) data for model training. We compiled GS cofunctional gene pairs from the following two independent biological processes or pathway annotations: UniProt Gene Ontology biological processes (UniProt-GOBP) (24) and MetaCyc metabolic pathways (25). GS-positive gene pairs were generated by pairing genes annotated by the same biological process or pathway terms. Biased gold standard data can lead to biased network training (36). Thus, we excluded UniProt-GOBP terms that have more than 100 member genes as well as MetaCyc superpathway terms. We compiled 19,899 and 6,989 gene pairs that share annotations by the UniProt-GOBP and MetaCyc pathways, respectively, and then combined them to generate 25,526 GS-positive gene pairs, which covered 2,139 K. pneumoniae genes (40.2% of coding genes). To generate negative gold standard data, we used pairs of genes that were annotated by either the UniProt-GOBP pathway or the MetaCyc pathway but that did not share any annotation terms, which resulted in 2,261,065 GS-negative gene pairs.

### Benchmarking and integrating cofunctional links.

Cofunctional links inferred from different data sets initially come with data-intrinsic scores. These data-specific scores need to be recalibrated to be integrated with those from other data sets. Therefore, we previously developed a log-likelihood score (LLS) (22) based on a Bayes statistics framework. LLS values were calculated by the following equation:

$$\text{LLS}=1\text{n}\left(\frac{P(L|D)/P(\neg L|D)}{P(L)/P(\neg L)}\right)$$

where, for a given datum *D*, *P*(*L*|*D*) and *P*(*¬L|D*) represent the probabilities of the presence of GS-positive and GS-negative links, respectively, and *P*(*L*) and *P*(*¬L*) represent the probabilities of the presence of all GS-positive and GS-negative links, respectively. To avoid overfitting problems, 0.632 bootstrapping was used. This method is based on subsampling of the training data set with replacements to generate test data sets as described in our previous work (22). The final LLS value was equal to 0.632 × LLS_test_ + (1 – 0.632) × LLS_training_.

Since cofunctional links were found from various sources, a functional link was found to be supported by extensive evidence with respect to LLSs. However, the data were not independent, so the weighted sum (WS) method that was previously developed to take into account the correlation among multiple pieces of evidence were used (36). The WS is defined as follows:

$$\text{WS}=L_{0}+\sum_{i=1}^{n}\frac{L_{i}}{D\times i},\text{for all }L\geq T$$

where *L* indicates LLS, *L*_0_ represents the highest LLS among all supporting pieces of evidence, and *L*_i_ represents the remaining LLSs from data supporting the link ordered by rank *i*. *D* and *T* represent free parameters for the weight factor and LLS threshold, respectively.

### Cofunctional links between K. pneumoniae genes inferred from cocitation (KP-CC).

A cocitation approach (15, 16) was utilized to identify functional links between K. pneumoniae genes from PubMed articles. First, 3,420 Medline abstracts that included either “Klebsiella pneumoniae” or “K. pneumoniae” were collected. Then, the significance of the presence of two K. pneumoniae genes (identified by the use of either systematic or common names) that appeared together in the same article was calculated by the one-tail Fisher’s exact test. Inferred cofunctional links were ordered by decreasing –log(*P* value), and then the LLS was calculated for every bin of 1,000 gene pairs from the most significant one. We found a sigmoid regression model for the relationship between −log(*P* value) and LLS and then assigned LLS values for individual gene pairs using the regression model.

### Cofunctional links between K. pneumoniae genes inferred from similarities in protein domain profiles (KP-DP).

Protein domains are functional and evolutionary units of proteins. Therefore, proteins that are functionally associated tend to share similar domains. We previously developed a method to infer cofunctional links between coding genes based on similarities between domain profiles (17). Domain occurrence profiles of proteins were generated based on data in the InterPro database (37). The similarities of domain profiles between proteins were measured based on weighted mutual information (WMI) (17), in which rarer domains were weighted more for calculating the mutual information (MI) score. Next, we found the nonlinear regression between WMI and LLS, which was later used to assign LLS values to individual gene pairs.

### Cofunctional links between K. pneumoniae genes inferred from phylogenetic profiles (KP-PG) and gene neighborhood (KP-GN).

Cofunctional links between K. pneumoniae genes were inferred based on two types of genomic context information: phylogenetic profiles (PG) and gene neighborhood (GN). In this study, we used a total of 1,747 (122 archaea and 1625 bacteria) genomes.

During speciation, functionally associated genes are likely to be retained together or lost together. Therefore, cofunctional links can be inferred from the coinheritance pattern across many species. We first performed a BLASTP search of all K. pneumoniae protein-coding genes against 1,747 genomes. Phylogenetic profiles of K. pneumoniae protein-coding genes were built on the basis of the best hit score and the −log(E value) for each genome. The association of two phylogenetic profiles was then measured by calculating the MI score as described in our previous work (20). We previously found that we were able to construct a better cofunctional network using phylogenetic profiles that are specific for each domain of life (*Archaea*, *Bacteria*, and *Eukarya*) (19). Two networks were inferred from a profile based on 122 archaeal genomes and from a profile based on 1,625 bacterial genomes and were then integrated into a single network.

We also inferred the cofunctional relationships between genes based on their genomic neighborhood. For example, genes encoded in bacterial operons are cotranscribed and generally participate in the same biological process. Even eukaryotic genes, orthologs of which have neighborhood relationships in bacterial genomes, tend to be functionally associated. We inferred cofunctional links based on the gene neighborhood across the 1,747 archaeal and bacterial genomes with two complementary measures of gene neighborhood: distance-based measures and probability-based measures (18). In addition, a metagenome-based gene neighborhood method (38) was used for KlebNet. We utilized contig sequences from the Human Microbiome Project (39) and TARA Ocean Project (40). All protein-coding genes of K. pneumoniae were aligned to the contigs by the use of DIAMOND software (41).

### Cofunctional links between K. pneumoniae genes by orthology-based transfer from other species (associalog).

Because pathways are conserved between species, we can infer functional links between genes by transferring the functional association between orthologous gene pairs from other species. We previously named such evolutionarily conserved gene pairs “associalogs” (21). We inferred cofunctional links between K. pneumoniae genes from cofunctional networks previously constructed for three other microbial organisms: Escherichia coli (EcoliNet) (42), Pseudomonas aeruginosa (PseudomonasNet) (43), and Saccharomyces cerevisiae (YeastNet) (44). We could transfer cofunctional links based on the cocitation between E. coli genes (EC-CC), P. aeruginosa genes (PA-CC), S. cerevisiae genes (SC-CC); links based on coexpression (45) between E. coli genes (EC-CX), P. aeruginosa genes (PA-CX), and S. cerevisiae genes (SC-CX); and links based on literature-curated protein-protein interactions between E. coli genes (EC-LC) and P. aeruginosa genes (PA-LC). In addition, we inferred functional links from high-throughput protein-protein interactions between Mycoplasma pneumoniae genes and Staphylococcus aureus genes. We mapped orthologous relationships between genes using Inparanoid software, which takes in-paralogs into account (46).

### Complementation assay.

To determine whether the putative candidates had any role in meropenem resistance, the genes were complemented using ZpUC19 plasmid (a gift from Y. Suzuki at the J. Craig Venter Institute, USA) (47). The plasmids were then introduced into electrocompetent cells as previously described (48), and the electroporated cells were recovered in low-salt LB broth (Sigma-Aldrich, St. Louis, MO, USA) and selected on low-salt LB agar plates carrying 1,000 μg/ml of zeocin. The complemented strains were confirmed using M13-pUC19_F and M13-pUC19_R primers. The complemented strains were then grown overnight in 5 ml of LB broth at 37°C with continuous shaking. On the following day, the strains were inoculated into fresh LB broth, and the optical density at 600 nm (OD_600_) was measured at 0, 1, 2, 3, 4, 5, 6, 8, 12, and 24 h. The values were plotted using GraphPad Prism 5.01 for Windows (GraphPad Software Inc., San Diego, CA, USA). The experiments were carried out in triplicate.

### Fitness assay in Galleria mellonella larvae.

G. mellonella larvae were purchased from SWorm Ltd., South Korea, and were used within 5 days of receipt. The bacterial strains were grown in low-salt LB broth for 3 h and centrifuged at 4,000 × *g* for 20 min at room temperature. The obtained pellets were washed once and resuspended in phosphate-buffered saline (PBS) buffer to obtain the required concentration. The larvae were injected with 10 μl of the bacterial cells (OD_600_ of 1.0) into the last left proleg using a Hamilton syringe and incubated at 37°C inside a petri dish. Control strains were maintained by injecting 10 μl of PBS buffer. Rates of survival of the larvae were recorded every 24 h up to 4 days. Similarly, at 4-h postinfection, the larvae were injected with a single dose of 60 mg meropenem/kg of body weight, and the survival rate was monitored every 24 h up to 4 days.

### Data availability.

The gene expression data sets generated during the current study are available in the Gene Expression Omnibus (GSE115539). Genome sequencing short-read data for K26 and K56 strains were deposited in SRA (SRP150097).
